# Mental Well-Being of Older People in Finland during the First Year in Senior Housing and Its Association with Physical Performance

**DOI:** 10.3390/ijerph15071331

**Published:** 2018-06-25

**Authors:** Sinikka Lotvonen, Helvi Kyngäs, Pentti Koistinen, Risto Bloigu, Satu Elo

**Affiliations:** 1 Research Unit of Nursing Science and Health Management, Medical Research Center of Oulu, University of Oulu, P.O. Box 5000, 90014 Oulu, Finland; helvi.kyngas@oulu.fi (H.K.); satu.elo@oulu.fi (S.E.); 2 Research Unit of Nursing Science and Health Management, Medical Research Center of Oulu, Oulu University Hospital, Kajaanintie 50, 90220 Oulu, Finland; 3 Faculty of Medicine, University of Oulu, P.O. Box 5000, 90014 Oulu, Finland; pentti.koistinen@outlook.com; 4 Medical Informatics and Statistics Research Group Oulu, University of Oulu, P.O. Box 5000, 90014 Oulu, Finland; risto.bloigu@oulu.fi

**Keywords:** mental well-being, older people, relocation, senior housing, physical performance, physical activity

## Abstract

Growing numbers of older people relocate to senior housing, when their physical or mental performance declines. The relocation is known to be one of the most stressful events in the life of older people and affect their mental and physical well-being. More information about the relationships between mental and physical parameters is required. We examined self-reported mental well-being of 81 older people (aged 59–93, living in northern Finland), and changes in it 3 and 12 months after relocation to senior housing. The first measurement was 3 months and the second measurement 12 months after relocation. Most participants were female (70%). Their physical performance was also measured, and associations between these two were analyzed. After 12 months, mental capability was very good or quite good in 38% of participants, however 22% of participants felt depressive symptoms daily or weekly. Moreover, 39% of participants reported daily or weekly loneliness. After 12 months participants reported a significant increase in forgetting appointments, losing items and difficulties in learn new things. They felt that opportunities to make decisions concerning their own life significantly decreased. Furthermore, their instrumental activities of daily living (IADL), dominant hand’s grip strength and walking speed decreased significantly. Opportunities to make decisions concerning their life, feeling safe, loneliness, sleeping problems, negative thoughts as well as fear of falling or having an accident outdoors were associated with these physical parameters. In addition to assessing physical performance and regular exercise, the various components of mental well-being and their interactions with physical performance should be considered during adjustment to senior housing.

## 1. Introduction

Growing numbers of older people are relocating to senior housing, when their physical or mental performance declines and their living arrangements are no longer appropriate [1,2]. The relocation is caused by various factors: an inability to remain at home, poor health and physical performance, loneliness, depression, not wanting to be a burden to the family, the need for services that support independent living, barrier-free environments and safety [3,4]. In Finland, such independent living communities comprise rental properties built especially for older people with functional limitations and are usually owned by national housing providers, non-profit corporations or foundations. Rents are market driven and vary with location, amenities (such as gym, cafe or swimming pool) and services provided [5]. The residents pay for rent and services. Those with low income have the possibility to apply for rent allowance, a form of social security paid by the social insurance institution of Finland. Moreover, municipality gives income related service vouchers for residents with a regular need of services. Senior housing services such as home care and healthcare and physical and social activities enable older people to continue to live independently in a supportive environment with limited assistance [6]. Most senior housing residents are older women, who don’t need assistance 24/7, but may benefit from a senior friendly environment with supportive services and increased social opportunities [7,8]. In Finland municipalities also offer nursing homes and institutional care for seniors who need around-the-clock assistance and care daily. The difference between senior housing and nursing home is that senior housing residents are independent and able to make decisions. Nursing homes are similar to institutional care; residents are no longer able to take care of themselves.

Research has found both positive and negative dimensions of relocation to senior housing and the new environments present both challenges and opportunities [7,9,10]. Leaving the home and adjusting to a new social environment is one of the most stressful events in the life of older people [3,11,12], affecting mental, physical and social well-being [4,6,11,13]. The relocation process is linked to physiologic and psychosocial disorders such as the relocation stress syndrome, which has been accepted as a nursing diagnosis by the North American Nursing Diagnosis Association classification scheme since 1992. The relocation stress syndrome is a disorder where individuals experience difficulty coping with the familiar environment to unfamiliar [14]. Rossen and Knafl [6] found that 4 months after relocation into senior housing, 45% of older women experienced a positive, healthy and successfully transition but more than half experienced only limited integration. In one review which determined factors that influenced relocation transitions, some new residents described relief of household duties, meeting new friends, improved safety, increased socialization and continuous health supervision as benefits of relocation. However, an equal number of residents experienced stress, anxiety, depression, helplessness and fear [10]. An active role in decision making and opportunities to exercise choice are associated with successful adjustment [15]. 

Experiencing functional limitations may precipitate the move to senior housing [16]. It follows that older people relocating in to senior housing may perform worse in physical performance measures, have more health problems and frailty than the community dwelling peers [8,17,18]. Besides, relocation has been related to a decline of physical performance [6,14] and increased limitations of instrumental activities of daily living (IADL) [19]. However, physical performance and physical activity are considered important to mental well-being and cognitive functions in later life [20,21,22]. A longitudinal study showed that an increase of 1 point on the physical activity measure was associated with nearly a 20% reduction in the probability of becoming depressed at the 5-year follow up [23]. Likewise, the incidence rate of dementia has been shown to be significantly lower among older people who exercised 3 or more times a week [24].

Older peoples’ mental well-being is a combination of multiple psychological social and biological factors, including cognitive ability mood, social relationships, independence and safety [25]. The changes that often come in later life can decrease mental well-being. Many older people lose their ability to live independently because of limited physical performance or cognitive functions, frailty or poor health conditions [26]. Older people are also more likely to experience deaths of loved ones and relocation to new environments. Furthermore, dementia and depression among the elderly are common and as people age they are more likely to experience several conditions at the same time [25]. Sleeping problems also become more common with increasing age, health decline, depression and memory loss [27].

Environmental stressors influence older peoples well-being [7]. Previous research has shown a major life change as relocation affects mental and physical functioning [4,6,11,13]. Moreover, older people are relocating to senior housing when their mental and physical functioning declines [8,17,18]. It is known that physical activity and physical performance are extremely important to mental well-being in a new environment [23,24]. Likewise, the home and living environment have important relevance in supporting mental and physical wellbeing of older people [7]. Knowledge of mental wellbeing and physical performance of senior housing residents is scarce. Our hypothesis is, that self-reported mental-wellbeing of older people is associated with measured physical performance during the first year in senior housing. To our knowledge, no previous studies have examined the interaction between mental well-being of older people and physical performance during the first year in senior housing. Thus, there is need to examine older peoples’ mental well-being and its’ association with physical performance after a major life change like relocation to senior housing. This study aims to evaluate older people’s self-reported mental wellbeing and associated changes during the first year in senior housing and links to their physical performance. 

## 2. Materials and Methods 

### 2.1. Study Design and Data Collection

Our data are from a longitudinal study, recording participants’ self-reported mental well-being and measuring their physical performance 3 and 12 months after relocation to senior housing. The participants were, 81 older people, born between 1922 and 1956 (Table 1), who had moved to one of the 11 senior housing facilities owned by three private organizations, in Oulu, northern Finland. This study is quantitative. The qualitative data from the interviews will be presented in the next publication. Three months baseline was selected, because both emotional and physical responses attributed to the relocation stress may occur strongest during first 3 months after moving in new senior housing environment [18].

Data collection involved face to face structured interviews, lasting 2-h, during home visits 3 and 12 months after moves to senior housing between June 2014 and December 2015. The data was collected in different seasons. The inclusions criteria were: relocation to senior housing 3 months before the first interview and measurement round; capacity to understand the procedures and aims; willingness to participate; answer the multiple-choice questions reliably (did not have memory disorder) and engage in the physical performance measurements. An information letter was sent to all older people who had moved to senior housing 3 months before (*n* = 121), then they were telephoned to ask whether they would participate. At the end of this phase, 34% of the target group were excluded: 22 declined and 18 people did not meet inclusion criteria. The first data collection 3 months after relocation consisted of 81 older people of which 70% were women. In the second data collection 12 months after relocation 71 people of the first sample were re-interviewed and measured. The sample is typical of older people who relocate to senior housing in Finland, excluding residents with memory disorders [18]. In this study, senior houses were located in an urban area near services (such as shops, food store, health center, etc.). Most of the residents had moved into senior houses within the same city in Northern Finland.

### 2.2. Instruments

#### 2.2.1. Mental Well-Being

Participants’ self-reported mental well-being data were collected using the Oldwellactive questionnaire, developed as a tool to elicit older peoples’ self-rated perceptions of their well-being and wellness [28]. The Oldwellactive wellness profile consist of nine domains and 75 multiple-choice variables, operationalizing and assessing the well-being of older people living at home. It was constructed, piloted and validated in elderly home care services in Oulu, Finland [28]. In this study, we used a modified Oldwellactive questionnaire to assess participants perceptions of their mental well-being 3 and 12 months after relocation. The assessment consisted of 26 items inviting Likert-type responses with 5 alternatives on the Likert scale. Mental capability was measured by nine questions (Table 2), mood by eight questions (Table 3), loneliness by 3 questions (Table 4) and safety by six questions (Table 5). These multiple-choice variables were chosen because they operationalize and assess the self-reported mental well-being of older people in the Oldwellactive wellness profile. These factors are also mentioned in the previous research as risk factors for mental well-being of older people [25]. The response formats are provided in the tables.

#### 2.2.2. Background Characteristics and Physical Performance 

Likewise, data on participants’ background characteristics, IADL performance, grip strength and lower body strength were collected using the Oldwellactive questionnaire [28]. Their IADL-performance was measured using 11 items from the previously published IADL-scale [29], asking “Do you cope independently with the following tasks?” (heavy housework, outdoor activities, shopping, finances, medicines, cooking, dressing, personal hygiene, bathing, using the toilet). Three answers were available: yes (2 points), yes, but I have difficulties (1 point) and no (0 point). The range of total scores was between 0–22 and related to the participants’ self-rated independence in IADLs. Lower body strength, grip strength and walking speed were measured to assess participants’ physical performance. Lower body strength was measured by counting the number of full stands older people could do in 30 s [30] from a straight-backed chair without arms (seat was approximately at knee height). The chair was placed against a wall to prevent it from moving. The participant was instructed to rise, after the signal was given, stand up and return back to fully seated position. The score was the total number of full stands without the use of hands. Grip strength of the right and left hand was measured to the nearest kg using a dynamometer (Jamar, Lafayette, Indiana, USA) which participants were asked to squeeze as hard as possible. Walking speed was evaluated by one of the three components of the Short Physical Performance (SPPB) test [31]. Each participant was asked to walk at their usual speed across a 4 m course marked on the floor using carpenters tape, with 60 cm buffer zones at both ends. The time required to walk the 4 m at usual speed was recorded. This test was repeated twice, and the fastest time was used for analysis. Participants wore walking shoes and used walking aid if needed. SPPB scores were not used for further analysis, except for the time in seconds to complete the 4 m course. 

### 2.3. Statistical Analysis

Analyses were undertaken using SPSS Statistics 18 (SPSS Inc., Chicago, USA) [32]. Frequencies, percentages and means represented baseline characteristics. The nonparametric Marginal Homogeneity test was used to indicate differences in 3 and 12 months self-reported mental wellness. New variables were calculated to indicate change in self-reported mental wellness. The paired samples *t*-test was used to compare means of the physical performance measurements at 3 and 12 months. New variables were calculated to indicate changes in physical performance measurements. The One Sample *t*-test was used to examine the differences between the physical performance measurements. The Non-parametric Wilcoxon test was used to compare differences between physical performance measurements when the data distribution was not normal. The Independent Samples *t*-tests and nonparametric Mann Whitney U-tests were used to test associations between self-reported mental factors and change in measured physical performance. A *p*-value of 0.05 was set as the threshold for statistical difference.

### 2.4. Ethical Issues

Permissions (29042014, 01092014, 02092014) to proceed were received from the three organizations that maintain the facilities. An information letter was sent to potential participants and followed up as described above. In order to ensure that participants were able to give informant consent the participants with memory disorder were excluded. All home visits and data collection were performed by the same interviewer: a physiotherapist with experience of home interviews and physical performance measurements. All participants received written information about the study, contact information for further questions and signed a statement of informed consent. All instruments used have been designed and validated for assessing self-rated perceptions or measuring the performance of older people.

## 3. Results

### 3.1. Background Characteristics

The subjects (*n* = 81) were 59–93 years old; the mean age was 81 years (*SD* = 7.71); most (70%) were female; 75% lived alone; and 72% used home care or personal care services (Table 1). The three most common medical conditions were coronary heart disease, musculoskeletal disease and neurological disease. Almost three quarters (72%) considered their state of health to be moderate. 

### 3.2. Self-Reported Mental Capability 

After 12 months, mental capability (ability to think clearly, memory) was very good or quite good in 36% of participants (Table 2). Almost one quarter (23%) reported that their mental capability had decreased, 17% reported an increase and 61% no change. However the change in self-reported mental capability was not statistically significant during the first year in senior housing. Reports of not forgetting appointments or losing items decreased significantly (*p* = 0.025), from 69% to 58% during the first year in senior housing. Moreover, the participants difficulties to concentrate rose almost significantly (*p* = 0.068). After 12 months 25% of older people reported that difficulties to consentrate increased, 13% reported decrease and 62% reported no change. Reports of no difficulty in learning new things and skills decreased significantly (*p* = 0.016), from 31% to 21% after 12 months. In the second round of data collection, almost all participants (96%) said they followed current affairs and events (in TV, radio, newspaper or internet) and most of them (63%) had no difficulty in concentrating.

### 3.3. Self-Reported Mood 

After 12 months 22% of participants felt depressive symptoms daily or weekly and 59% rarely or never (Table 3). There was no statistically significant change in depressive symptoms during the first year in senior housing. Reports of having opportunities to make decisions about life decreased statistically significantly during the first year in senior housing (*p* = 0.018), falling from 82% after 3 months to 69% after 12 months. Still, after 12 months, the most frequently expressed feelings were that respondents always had such opportunities (69%) and they rarely had difficulties in controlling negative feelings or aggressions, while talking with other people (56%) and they rarely felt distressed or anxious (53%). After 12 months 39% of participants reported that they had no or few problems sleeping, whilst 31% reported many or constant sleep problems. At this time 44% reported that they were very or quite positive about the future, almost half reported neither positive nor negative thoughts and 8% reported quite or very negative thoughts.

### 3.4. Self-Reported Loneliness 

After 12 months 60% of participants reported no or only a little loneliness. Moreover, 39% of participants reported daily or weekly loneliness, 14% monthly and 47% rarely or never (Table 4). However, feeling lonely increased almost significantly (*p* = 0.084). Thirty percent of older people reported feeling more often lonely, 20% less often and almost half reported no change. Fourteen percent of participants said they had no one to talk to about personal affairs daily or weekly, 44% monthly or rarely and 42% never. There was no significant change in self-reported loneliness during first year in senior housing. The most frequently expressed feelings 12 months after relocation were never or rarely feeling there was no one to talk about personal affairs (82%) and suffering little or no loneliness (60%). 

### 3.5. Self-Reported Safety

The feeling that life is safe significantly increased (*p* = 0.046) while living in senior housing. Percentages perceiving life to be very safe rose from 20% after three months to 42% after 12 months (Table 5). Being afraid of sudden illness while at home statistically significantly decreased (*p* = 0.046). Being very much or quite a lot afraid of sudden illness while at home decreased from 13% to 3%. There were no statistically significant changes in other items of self-reported safety, as can be seen in Table 5. 

### 3.6. Measured Physical Performance

During the first year IADL scores (*p* = 0.002) dominant hand’s grip strength (*p* = 0.033) and walking speed (*p* = 0.002), all decreased significantly (Table 6). IADL scores by 6%, mean grip strength of right and left hands decreased by 7.7 and 2.2%, respectively, mean walking speed by 22% and mean number of chair-stands in 30 s also decreased slightly, but not significantly (*p* = 0.150). Moreover, the dominant hands grip strength decreased 12% in the group of 55–74 year old participants, 21% in the group of 75–84 year old and 23% in the group of 85–94 year old. Likewise, IADL-scores decreased 10% in the group of 55–74 years old participants, 21% in the group of 75–84 years old and 10% in the group of 85–90 years old. The 4 m usual walking speed decreased 4% in the group of 55–74 year old participants, 25% in the group of 75–84 years old and 14% in the group of 85–94 years old.

### 3.7. Associations between Participants’ Perceptions of Their Mental Well-Being and Measured Physical Performance

Mean IADL-scores decreased more among participants who reported decreases in opportunities to make decisions about their life (*M* = 2.08, *SD* = 2.5, *n* = 12) than among those who reported such opportunities increased or remained the same (*M* = 0.58, *SD* = 2.0, *n* = 59). An independent samples *t*-test indicated that this difference was statistically significant *t*(69) = −2.2, *p* = 0.03. Moreover, mean IADL scores decreased more among participants who reported more negative thoughts (*n* = 15) about the future than among those who reported more positive or neutral thoughts about the future (*n* = 56). A Mann-Whitney U-test indicated that this difference was statistically significant (*U* = 263, *Z* = −2.26, *p* = 0.024). Mean IADL scores also decreased more among participants who reported feeling less or equally safe (*M* = 1.12, *SD* = 1.69, *n* = 49) than among those who reported increased feelings of safety (*M* = 0.18, *SD* = 2.3, *n* = 22). An independent samples *t*-test indicated that the difference was statistically significant *t*(69) = 1.2, *p* = 0.053.

Mean walking speed decreased more among older people who reported more or unchanged loneliness (*n* = 49), than among those, who reported less loneliness (*n* = 16). A Mann-Whitney U test indicated that this difference was statistically significant (*U* = 534, *Z* = 2.16, *p* = 0.031). Moreover, mean walking speed decreased more among older people who reported more or unchanged sleeping problems (*n* = 56), than among those, with reduced sleeping problems (*n* = 9). A Mann-Whitney U test indicated that this difference was statistically significant (*U* = 413, *Z* = 3.07, *p* = 0.002).

Right hands grip strength decreased more among participants who reported more fear or unchanged fear of falling or some other accident while moving outdoors (*M* = 2.77, *SD* = 7.1, *n* = 53), than among those, who reported reduced fear while moving outdoors (*M* = −1.28, *SD* = 4.4, *n* = 18). An independent samples *t*-test indicated that this difference was statistically significant *t*(69) = 2.2, *p* = 0.028).

## 4. Discussion

It is known that relocation is a major life change and that older people are at risk of developing relocation stress syndrome, leading to increased confusion when moving to a new environment. [10,13,14]. According to a previous study, relocation has little effect on cognitive abilities measured by Mini-mental State Examination (MMSE) [33] but if residents may have even mild undiagnosed cognitive impairment, the process can become more challenging in combination with memory difficulties [14,34]. In the present study almost one quarter of participants reported decrease of mental capability. It is possible that mild cognitive impairment or undiagnosed early stage dementia is the reason for relocation of some residents: indeed, mild cognitive impairment affects 4% to 19% of people aged 65 years or older [35].

Physical activity and exercise are the most important factors for the maintenance of cognition, independence and physical performance as well as being considered important for positive psychological functioning [22,35]. Its importance may increase after relocation to senior housing [6,14]. After 12 months, most of the participants reported following current affairs and events (via TV, radio, newspaper or internet). A previous study found that senior housing residents were commonly involved in watching TV or listening to the radio (these are common recreational activities among older people) in their own apartments [36]. Even though, following the news is important, there is a risk that older people spend most of their time in their apartments and miss out on activities and interaction with others. Close contacts and nursing staff should pay attention to residents, who remain isolated in their apartments, and should encourage them to participate in common activities, particularly because lifestyle factors such as social isolation and physical inactivity both increase the risk of developing dementia [35].

Participants’ perceptions that they have the opportunity to make decisions concerning their life decreased statistically significantly during the first year in senior housing. Independent decision-making enables people to exercise control over their own life and its importance and impact may increase with age [37], and when the experience of aging necessitate relocation to new environments [34]. The amount and quality of control as well as having an active role in the decision-making process are both related to older peoples’ positive adjustment to new environment [10,15]. Older people who feel that they have limited control over their activities and that their control is over-ridden by staff or people close to them, experience difficulties adjusting to senior housing and feel powerless to improve their situation [3].

In the present study, more than half of the participants suffered little or not at all from loneliness. However, feeling lonely increased almost significantly during the first year in senior housing. One quarter reported increased and almost a quarter decreased loneliness. Most of the participants lived alone and were widowed or divorced. Loneliness is higher among women and widowhood is a main predictor of loneliness [38,39]. Studies have also shown that loneliness increases with age and is associated with poorer physical and mental health [40,41]. It is known that loneliness, social isolation and depression interact [40]. Relocation to senior housing provides an opportunity for new social interactions and friends, and women who participate in social activities do make new friends and integrate better into senior housing. Less social women, who have strained and few connections with family and friends remain without friends in senior housing, have difficulties integrating, limit their social contacts at meal times and do not participate in activities [42]. Staff and close contacts should recognize socially isolated, lonely and depressive residents and encourage them to get together, make new friends and participate in social activities. Joining activities and getting to know neighbors supports healthy transitions [10,42].

We found that feeling that life is safe increased significantly during the first year in senior housing. Moreover, being afraid of an attack of sudden illness while at home significantly decreased. In reviews of older peoples’ experiences with residential care placements those involved appreciated safety of their new environment and felt more secure [10,12]. Improved security and assistance also encourage relocation to senior housing [4]. Senior houses are built barrier free (no thresholds, automatic doors, elevators, handrails, non-slip floors e.g.,) for older people with reduced physical performance and should be suitable for moving around with walking aids and wheelchairs. Senior facilities offer support services and assistance in a sheltered environment where older people can continue independent living, furthermore there is the possibility to get safety monitors with emergency buttons that offers alert in case of falls or illness.

In our study, measured physical performance was poorer than average for the home-living population of the same age [30,43,44]. Furthermore, dominant hand grip strength, IADL-performance and walking speed significantly decreased during the first year in senior housing [18]. For comparison, in the meta-analysis, that obtained average grip strength of older people, right hand strength was 24 kg and left 22 kg [43]. Furthermore, according to the study of physical performance of 79–89 years old Finnish war veterans, within these right hands grip strength was 29 kg and left hand 26 kg [45]. In an American study, 30 s chair stand mean score was 13 among older people [30]. Moreover, the 30 s chair stand cutoff point of 8 or less (30 s chair stands ≤8) identifies risk of functional decline within 60–94 years old people [46]. In the previous study examining decline of walking speed of 60–89 years old people, the usual walking speed was 1.1 m/s and it declined −0.003 m/s in one year [44]. Furthermore, based on previous study, almost half of 65–85 year old people are categorized as independent to carry out IADL, whereas 5.5% are dependent for all IADL [47]. The previous study has shown that older people living in senior housing are less physically active and have lower physical performance than those of the same age living in the community [16]. Furthermore, more physically active senior housing residents have fewer functional limitations at a 10 months follow-up than less active individuals [8]. Senior housing should provide an environment tailored to the needs of older people, whose physical performance will gradually decline [18]. When older people’s physical performance does decline, they want to make their lives easier and move to smaller dwellings closer to services, but this may also reduce their daily activities, amounts of physical activity and physical performance [16]. The negative effect of relocation on physical performance, should be buffered by muscle strength and balance training groups [8] and social support from close contacts and staff [19]. The musculoskeletal diseases were common among the participants (73%) (Table 1), but we didn’t explore to what extend these might have affect to physical performance among first year in senior housing.

IADL performance decreased significantly more among participants reporting decreases in opportunities to make decisions about their life, and among individuals who reported having more negative thoughts about the future. The need for assistance with IADLs often necessitates relocation to senior housing that offers supporting services. However, one of the primary needs of older people is to maintain autonomy and decision making in their new environment: thus, supporting choice-driven decisions and independency is crucial [48]. In our study, IADL decreased more among participants who felt less safe. Probably most frail and depressive old people are at major risk of IADL decline even in barrier-free senior housing. It is important to consider the various needs of residents with varying levels of mental and physical capacity as well as to assess and support their IADL-performance and physical activity [19,36]. Listening, offering choices and promoting decision making by residents during home and healthcare service planning, should occur in the context of the feelings and wishes of the individuals and family members involved [14].

Mean walking speed decreased more among older people who reported more or unchanged loneliness than among those who reported less loneliness. This finding is supported in other studies showing that mobility problems are associated with older women’s loneliness [39,49]. In our study participants had poor extremity function, and this has been shown to be associated with depressive older women’s social participation [50]. Moreover, lack of company for outdoor activities is associated with walking difficulties [51] and has been shown to be a significant barrier to older people’s physical activity (while supportive and motivating company promotes it) [52]. Opportunities to walk in guided groups could encourage lonely residents to participate in physical activity with other residents, facilitate making new friends and support their ability to cope in their new environment. Likewise, mean walking speed decreased more among older people who reported more or unchanged sleeping problems than among those who reported fewer sleeping problems. In our study almost one quarter of participants often or always had sleeping problems and almost half sometimes experienced such problems. A recent study has shown that poor sleep plus daytime sleepiness is related to walking speed and self-reported balance [53,54]. Sleep disorders are common among older people [27], related to mobility problems and falls [54]. Similarly, recreational walking is associated with fewer sleep difficulties [55]. Friedman et al. [55] showed that an eight-week program involving group sessions to identify dimensions of mental well-being reduced older peoples’ sleep problems and depression. Sleepiness during day time can result in falls, reduce physical activity and cause a decline in physical performance. Sleep assessment of residents is needed after relocation to senior housing and recreational walking, social and physical activity group activities should be used to reduce sleep disorders and mobility problems.

Right hand grip strength decreased significantly more among participants who reported more or unchanged fear of falling or having some other accident while moving outdoors than among those who reported less fear. Grip strength is known to reflect general muscle strength and recent studies have shown weak grip strength is associated with fear of falling [56,57]. In our study participants lived in northern Finland where outdoor mobility is challenging in winter. A previous study showed that older people with poorer extremity performance, perceiving snow and ice as barriers and reporting fear of falling and insecurity while walking outdoors, suffered increased walking limitations [58]. Regular muscle strength and balance training, after relocation to senior housing, are very important for older people with weak muscle strength and who fear of falling outdoors.

Feelings of fear can be reduced by regular balance and muscle strength training. Various kinds of gerontechnology (moving aids, non-slip-shoes, safety with emergency buttons, mobile phones) can also help older people to overcome fear of falling or sudden illness associated with their restricted physical performance outdoors. Fear of falling or sudden illness can also be reduced by home care staff and close contacts who should encourage residents to participate in exercise groups and physical activity and introduce them to appropriate gerontechnology. Programs to assess and promote mental well-being and physical performance of residents are needed. For example, regular assessment of mental and physical capacities, together with regular exercise designed to maintain muscle strength and balance are important to minimize decline of physical performance. Social group activities should be designed for needs of residents with varying mental, physical, social and capacity. Furthermore, it is extremely important that staff and close people understand new residents’ psychological, physical and social challenges and facilitate adaptation to the new environment. Training for staff to promote residents’ mental well-being and physical activity is also required. Future studies with larger samples could clarify effects of physical activity on mental well-being of senior housing residents.

### Strenghts and Limitations

The strength of the study is that we used both self-reported and measured information to evaluate older people’s mental well-being and its association with physical performance. Interviews and measurements were conducted by the same person, who was a physiotherapist and had experience of the instruments used and home visits. This enhanced the validity of study. The Oldwellactive instrument has strengths in providing means to illustrate wellness-related dimensions of mental- wellbeing via an interviewee-centered approach [28]. One limitation is that our findings are based on quite a small sample of older people, mostly women. However, in other studies of people living in senior housing, the participants were also women [7,8]. People with cognitive impairments were excluded from our sample, which limits generalization, but improves the reliability of older people’s own estimation of their mental well-being. It is also possible, that the frailest residents did not participate in the study, because of health problems. The participants lived in northern Finland and it should be noted that the data were collected in different seasons, and seasonal variations could have influenced the participants’ reported mental well-being and physical performance. Major limitation is also that we did not assess participants before moving in to senior housing because of the time schedule and human resources of the study. It is therefore unclear whether the changes observed are related to the move or are part of a longer-term process of age-related decline that was ongoing prior to the move. In addition, in the presentation of the association results, causality may have two-way effects. The causality is therefore unclear; for example, it is difficult to say does loneliness contribute to slower walking speed or does slow walking speed promote loneliness. The role of diseases on disability and physical performance decline in older people has been shown in previous studies [59]. In our study almost three quarters of participants had musculoskeletal diagnosis (arthritis, osteoporosis, sarcopenia, rheumatoid arthritis, artificial joints, amputation) and one quarter had neurological conditions (7% hemiplegia, 5%, Parkinson’s disease, 5% transient ischemic attack (TIA), 3% intracranial hemorrhage, 3% polyneuropathy, 2% mild cognitive impairment) (Table 1), but we did not explore what extend these have affected to changes in the mental capability.

## 5. Conclusions

The results of this study showed that older people’s self-reported mental capability and loneliness did not change significantly during the first year in senior housing. However, their impression that they forgot appointments and lost items increased and their feeling that they had no difficulties in learning new things and skills decreased significantly. In addition, their feeling of having opportunities to make decisions about their life decreased significantly. Further, feelings that life is safe, increased significantly. There were significant reductions in IADL-performance, dominant hand grip strength and walking speed. Furthermore, reductions in opportunities to make decisions and feeling that life is safe, were associated with reductions of IADL-performance. In addition, increase in loneliness and sleep problems were associated with reductions in walking speed and increased fear of falling, while moving outdoors was associated with reductions in dominant hand grip strength.

Relocation creates disequilibrium and confusion in older people’s lives. Thus, it is important to consider the experiences of older people with varying mental well-being and physical performance, not only those who can easily make new social contacts and participate in group activities. Residents’ active decision making and opportunities to make choices about their life are key factors in autonomy and successful adaptation to a new environment. Regular assessment of older people’s mental well-being and physical performance, together with regular social group activities, walking groups as well as muscle strength and balance training are important to minimize declines in mental and physical performance. Likewise, support for independency in IADL-activities in a new environment is extremely important. In particular, lonely, less social, depressive and physically inactive residents need encouragement to engage in both social and physical activities and make new friends. Family members, close contacts and staff play an important role in motivating older people to be socially and physically active and adapt to a new environment.

## Figures and Tables

**Table 1:** Background characteristics of participants 3 months after relocation to senior housing (*n* = 81).

Background Characteristics	*n*	%
**Age (years)**		
55–64	4	5
65–74	9	11
75–84	42	52
85–94	26	32
**Gender**		
Female	57	70
Male	24	30
**Housing**		
Lived alone	61	75
Lived with someone (cohabited)	20	25
**Marital status**		
Married	27	33
Unmarried	7	9
Widowed	30	37
Divorced	17	21
**Children**		
Has children	68	84
No children	13	16
**Medical conditions**		
Coronary heart disease	72	88
Musculoskeletal disease	59	73
Neurological disease	21	26
Diabetes	14	17
Respiratory organ disease	13	16
**Health**		
Very good	2	3
Quite good	10	14
Moderate	51	72
Quite bad or bad	8	11
**Financial situation**		
Very good	2	3
Good	23	28
Moderate	53	65
Poor or very poor	3	4
**Service use**		
No services	23	28
Services	58	72

**Table 2:** Self-reported mental capability of older people and changes in these variables 3 and 12 months after relocation to senior housing.

Mental Capability	3 Months		12 Months		Increased	Decreased	No Change	Significance *
	*n*	(%)	*n*	(%)	*n* (%)	*n* (%)	*n* (%)	*p*-Value *
*How do you perceive your mental capability? (ability to think clearly, memory)*								
Very good	3	(4)	3	(4)				
Quite good	31	(44)	23	(33)				
Moderate	30	(42)	42	(59)				
Quite bad	7	(10)	3	(4)				
Very bad	0	(0)	0	(0)				
	*M* 2.58	*SD* 0.73	*M* 2.63	*SD* 0.63	12 (17)	16 (23)	43 (61)	0.493
*Is it difficult for you to remember familiar names?*								
No	29	(41)	25	(35)				
Sometimes	34	(48)	41	(57)				
Often	8	(11)	5	(7)				
	*M* 1.70	*SD* 0.67	*M* 1.72	*SD* 0.59	14 (20)	13 (18)	54 (77)	0.862
*Do you forget appointments or lose items?*								
No	49	(69)	41	(58)				
Sometimes	19	(27)	25	(35)				
Often	3	(4)	5	(7)				
	*M* 1.35	*SD* 0.56	*M* 1.49	*SD* 0.63	5 (7)	15 (21)	51 (72)	0.025
*Do you find it difficult to learn new things and skills?*								
No	22	(31)	15	(21)				
Sometimes	42	(59)	42	(59)				
Often	7	(10)	14	(20)				
	*M* 1.79	*SD* 0.61	*M* 1.98	*SD* 0.64	8 (11)	20 (28)	43 (61)	0.016
*Do you find it difficult to concentrate?*								
No	48	(68)	45	(63)				
Sometimes	20	(28)	24	(34)				
Often	3	(4)	2	(3)				
	*M* 1.69	*SD* 0.57	*M* 1.83	*SD* 0.67	9 (13)	18 (25)	44 (62)	0.068
*Can you think as clearly as before?*								
No	14	(20)	13	(18)				
Sometimes	27	(38)	24	(34)				
Often	30	(42)	34	(48)				
	*M* 2.22	*SD* 0.76	*M* 2.29	*SD* 0.76	14 (20)	19 (27)	38 (54)	0.484
*Are you able to make decisions, choices or think through problems without assistance?*								
Always	39	(55)	37	(52)				
Most of the time	18	(25)	19	(27)				
Sometimes	6	(9)	5	(7)				
Rarely	7	(10)	8	(11)				
Not at all	1	(1)	2	(3)				
	*M* 1.77	*SD* 1.06	*M* 1.86	*SD* 1.14	11 (15)	18 (25)	52 (73)	0.473
*How often do you engage hobbies or other activities that keep your mind alert?*								
Daily	43	(61)	40	(56)				
Weekly	15	(21)	21	(30)				
Monthly	1	(1)	4	(6)				
Rarely	9	(13)	5	(7)				
Never	3	(4)	1	(1)				
	*M* 1.79	*SD* 1.22	*M* 1.68	*SD* 0.97	14 (20)	12 (17)	45 (63)	0.394
*How often do you follow current affairs and events? (on TV, newspapers, radio, internet)?*								
Daily	67	(94)	68	(96)				
Weekly	1	(1)	2	(3)				
Monthly	3	(4)	1	(1)				
Rarely	0	(0)	0	0				
Never	0	(0)	0	0				
	*M* 1.14	*SD* 0.62	*M* 1.06	*SD* 0.29	4 (6)	2 (3)	65 (92)	0.257

* According to the Marginal Homogeneity test.

**Table 3:** Self-reported mood and changes in these variables 3 and 12 months after relocation to senior housing.

Mood	3 Months		12 Months		Increased	Decreased	No Change	Significance *
	*n*	(%)	*n*	(%)	*n* (%)	*n* (%)	*n* (%)	*p*-Value *
*How often do you feel depressed?*								
Daily	6	(8)	5	(7)				
Weekly	11	(15)	11	(15)				
Monthly	7	(10)	13	(18)				
Rarely	38	(53)	34	(48)				
Never	9	(13)	8	(11)				
	*M* 3.46	*SD* 1.15	*M* 3.40	*SD* 1.10	14 (20)	17 (24)	50 (70)	0.663
*How often do you have difficulties in controlling your negative feelings, aggression or hate while talking with other people?*								
Daily	0	(0)	0	(0)				
Weekly	0	(0)	3	(4)				
Monthly	0	(0)	3	(4)				
Rarely	55	(77)	40	(56)				
Never	16	(23)	25	(35)				
	*M* 4.22	*SD* 0.42	*M* 4.22	*SD* 0.72	16 (23)	13 (18)	42 (59)	1.000
*How often do you feel distressed or anxious?*								
Daily	5	(7)	4	(6)				
Weekly	8	(11)	14	20)				
Monthly	7	(10)	8	(11)				
Rarely	41	(58)	38	(53)				
Never	10	(14)	7	(10)				
	*M* 3.60	*SD* 1.08	*M* 3.42	*SD* 1.09	15 (21)	28 (30)	35 (49)	0.209
*How often do you feel sad or hopeless?*								
Daily	10	(14)	12	(17)				
Weekly	9	(13)	7	(10)				
Monthly	13	(18)	12	(17)				
Rarely	37	(52)	36	(51)				
Never	2	(3)	4	(6)				
	*M* 3.16	*SD* 1.14	*M* 3.18	*SD* 1.22	16 (22)	13 (18)	42 (59)	0.926
*Do you feel that as you have become older you have become worthless?*								
Yes	17	(24)	21	(30)				
I don’t know	25	(35)	I8	(25)				
No	29	(41)	32	(45)				
	*M* 2.11	*SD* 0.76	*M* 1.95	*SD* 0.74	15 (21)	18 (25)	38 (53)	0.166
*Do you feel you have the opportunity to make decisions concerning your own life?*								
Always	58	(82)	49	(69)				
Most of the time	12	(17)	20	(28)				
Sometimes	1	(1)	1	(1)				
Rarely	0	(0)	1	(1)				
Never	0	(0)	0	(0)				
	*M* 1.21	*SD* 0.50	*M* 1.35	*SD* 0.58	3 (4)	12 (17)	56 (79)	0.018
*Do you have sleeping problems?*								
Not at all	20	(28)	17	(24)				
Rarely	18	(25)	11	(15)				
Sometimes	18	(25)	28	(39)				
Most of the time	10	(14)	9	(13)				
Always	5	(7)	6	(8)				
	*M* 2.46	*SD* 1.24	*M* 2.66	*SD* 1.21	9 (11)	24 (30)	38 (54)	0.118
*What sort of thoughts do you have about the future?*								
Very positive	2	(3)	2	(3)				
Quite positive	32	(45)	29	(41)				
Neither positive or negative	35	(49)	34	(48)				
Quite negative	1	(1)	5	(7)				
Very negative	1	(1)	1	(1)				
	*M* 2.53	*SD* 0.65	*M* 2.63	*SD* 0.72	11 (14)	15 (18)	45 (63)	0.237

* According to the Marginal Homogeneity test.

**Table 4:** Self-reported feelings of loneliness and changes in these variables 3 and 12 months after relocation to senior housing.

Loneliness	3 Months		12 Months		Increased	Decreased	No Change	Significance *
	*n*	(%)	*n*	(%)	*n* (%)	*n* (%)	*n* (%)	*p*-Value *
*Do you suffer from loneliness?*								
Not at all	21	(30)	25	(35)				
A little	19	(27)	18	(25)				
To some extent	21	(30)	17	(24)				
Quite a lot	9	(13)	9	(13)				
Very much	1	(1)	2	(3)				
	*M* 2.29	*SD* 1.07	*M* 2.22	*SD* 1.14	18 (25)	14 (20)	39 (55)	0.547
*How often do you feel lonely?*								
Daily	7	(10)	11	(15)				
Weekly	18	(25)	17	(24)				
Monthly	7	(10)	10	(14)				
Rarely	24	(34)	21	(30)				
Never	15	(21)	12	(17)				
	*M* 3.30	*SD* 1.32	*M* 3.08	*SD* 1.36	14 (20)	22 (31)	35 (49)	0.084
*How often do you feel you have no one to talk to about your personal affairs?*								
Daily	0	(0)	4	(6)				
Weekly	5	(7)	6	(8)				
Monthly	4	(6)	2	(3)				
Rarely	34	(48)	29	(41)				
Never	28	(39)	30	(42)				
	*M* 4.20	*SD* 0.84	*M* 4.06	*SD* 1.14	17 (24)	18 (25)	36 (51)	0.340

* According to the Marginal Homogeneity test.

**Table 5:** Self-reported feelings of safety and changes in these variables 3 and 12 months after relocation to senior housing.

Safety	3 Months		12 Months		Increased	Decreased	No Change	Significance *
	*n*	(%)	*n*	(%)	*n* (%)	*n* (%)	*n* (%)	*p*-Value *
*Do you perceive your life to be safe or unsafe?*								
Very safe	14	(20)	30	(42)				
Quite safe	56	(79)	37	(52)				
Nether safe or unsafe	1	(1)	3	(4)				
Quite unsafe	0	(0)	1	(1)				
Very unsafe	0	(0)	0	(0)				
	*M* 1.81	*SD* 0.42	*M* 1.64	*SD* 0.42	22 (31)	8 (11)	41 (58)	0.046
*Are you afraid of falling or having some other accident at home?*								
Not at all	37	(52)	38	(53)				
To some extent	19	(27)	1	(1)				
Quite a lot	5	(7)	19	(27)				
Very much so	10	(14)	13	(18)				
	*M* 1.83	*SD* 1.06	*M* 1.67	*SD* 0.80	18 (25)	14 (20)	39 (46)	0.192
*Are you afraid of a sudden attack of illness while at home?*								
Not at all	34	(48)	37	(52)				
To some extent	28	(39)	32	(45)				
Quite a lot	7	(10)	2	(3)				
Very much so	2	(3)	0	(0)				
	*M* 1.67	*SD* 0.77	*M* 1.50	*SD* 0.55	16 (22)	8 (11)	47 (33)	0.046
*Are you afraid of falling or having some other accident while moving alone outdoors?*								
Not at all	38	(53)	28	(39)				
To some extent	15	(21)	34	(48)				
Quite a lot	9	(13)	7	(10)				
Very much so	9	(13)	2	(3)				
	*M* 1.84	*SD* 1.07	*M* 1.76	*SD* 0.74	18 (25)	21 (30)	32 (46)	0.513
*Are you afraid of a sudden attack of illness while moving alone outdoors?*								
Not at all	46	(65)	49	(69)				
To some extent	20	(28)	18	(25)				
Quite a lot	3	(4)	4	(6)				
Very much so	2	(3)	0	(0)				
	*M* 1.45	*SD* 0.71	*M* 1.36	*SD* 0.59	14 (20)	10 (14)	47 (66)	0.273
*Are you afraid of the threat of violence or other disturbances while moving alone outdoors?*								
Not at all	45	(63)	47	(66)				
To some extent	22	(31)	22	(31)				
Quite a lot	3	(49	2	(3)				
Very much so	1	(1)	0	(0)				
	*M* 1.43	*SD* 0.64	*M* 1.36	*SD* 0.54	10 (14)	8 (11)	53 (75)	0.336

* According to the Marginal Homogeneity test.

**Table 6:** Measured physical performance parameters and associated changes 3 and 12 months after moving to senior housing.

Measured Physical Performance	*n*	3 Months	12 Months	3–12 Months	*95% CI*		*DF*	Significance *
		*Mean*	*Mean*	*Change*	*Lower*	*Upper*		*p*
*IADL scores*	71	14.65	13.82	−0.83	0.31	1.35	70	0.002
*Grip strength, right hand (kg)*	71	22.94	21.20	−1.74	0.14	3.35	70	0.033
*Grip strength, left hand (kg)*	71	20.80	20.28	−0.52	−0.73	1.77	70	0.410
*Usual walking 4 m (seconds)* *(Walking speed m/s)*	65	6.27	7.64	+1.37	−2.19	−0.53	64	0.002
		0.63	0.52	−0.11			70	
*No. of Chair stands in 30 s’*	71	6.58	6.13	−0.45	−0.16	1.06	70	0.150

* According to the Paired Samples *t*-test (two-tailed).
